# Supplementary material for: Genetic dissection of the different roles of hypothalamic kisspeptin neurons in regulating female reproduction
Source: eLife. 2019 Apr 4;8:e43999. doi: 10.7554/eLife.43999 (PMC6491090; doi:10.7554/eLife.43999)
Supplement: Transparent reporting form [file elife-43999-transrepform.docx]

***eLife’s* transparent reporting form**

We encourage authors to provide detailed information *within their submission* to facilitate the interpretation and replication of experiments. Authors can upload supporting documentation to indicate the use of appropriate reporting guidelines for health-related research (see [EQUATOR Network](http://www.equator-network.org/%20)), life science research (see the [BioSharing Information Resource](https://biosharing.org/)), or the [ARRIVE guidelines](http://www.plosbiology.org/article/info:doi/10.1371/journal.pbio.1000412) for reporting work involving animal research. Where applicable, authors should refer to any relevant reporting standards documents in this form.

If you have any questions, please consult our Journal Policies and/or contact us: [editorial@elifesciences.org](mailto:editorial@elifesciences.org).

**Sample-size estimation**

- You should state whether an appropriate sample size was computed when the study was being designed
- You should state the statistical method of sample size computation and any required assumptions
- If no explicit power analysis was used, you should describe how you decided what sample (replicate) size (number) to use.

Please outline where this information can be found within the submission (e.g., sections or figure legends), or explain why this information doesn’t apply to your submission:

The sample sizes are reported in the text and the statistical methods are described in the Materials and Methods section "*Data analysis and statistics*".

For electrophysiology, numbers are based on power analysis in which historic mean differences and standard deviations were used for a variety of data types (e.g., firing rate, synaptic transmission). A sample size of 10-18 cells/group depending on parameter has an 85% power to detect a difference between means of 30% with a significance level (alpha) of 0.05 (two-tailed) in an unpaired t-test. Numbers for electrophysiology were adjusted up by 15% to account for the reduced power of non-parametric tests needed for data that are normally distributed (for example all cells quiescent before treatment). From these calculations we established a range of 10-18 quality recordings per group.

For reproductive cycle assessment, no statistical methods were used to predetermine sample size. The numbers of samples in each group were based on those in previously published studies.

**Replicates**

- You should report how often each experiment was performed
- You should include a definition of biological versus technical replication
- The data obtained should be provided and sufficient information should be provided to indicate the number of independent biological and/or technical replicates
- If you encountered any outliers, you should describe how these were handled
- Criteria for exclusion/inclusion of data should be clearly stated
- High-throughput sequence data should be uploaded before submission, with a private link for reviewers provided (these are available from both GEO and ArrayExpress)

Please outline where this information can be found within the submission (e.g., sections or

figure legends), or explain why this information doesn’t apply to your submission:

This information is included for each experiment in the Materials and Methods section and the specific statistical analysis indicated in the text and in the method session "*Data analysis and statistics*".

Criteria for exclusion/inclusion of electrophysiological data is stated in the Materials and Methods section “*Whole-cell recordings.”* No outliners that met the inclusion criteria were excluded as they might represent the biological variance.

For reproductive phenotype experiments, animals that received stereotactic injections that were in non-targeted brain areas (assessed post hoc by immunohistochemistry staining) were excluded from the experiments.

**Statistical reporting**

- Statistical analysis methods should be described and justified
- Raw data should be presented in figures whenever informative to do so (typically when N per group is less than 10)
- For each experiment, you should identify the statistical tests used, exact values of N, definitions of center, methods of multiple test correction, and dispersion and precision measures (e.g., mean, median, SD, SEM, confidence intervals; and, for the major substantive results, a measure of effect size (e.g., Pearson's r, Cohen's d)
- Report exact p-values wherever possible alongside the summary statistics and 95% confidence intervals. These should be reported for all key questions and not only when the p-value is less than 0.05.

Please outline where this information can be found within the submission (e.g., sections or figure legends), or explain why this information doesn’t apply to your submission:

Statistical tests are introduced in the Materials and Methods, specified in the results, figure legends and tables.

(For large datasets, or papers with a very large number of statistical tests, you may upload a single table file with tests, Ns, etc., with reference to sections in the manuscript.)

**Group allocation**

- Indicate how samples were allocated into experimental groups (in the case of clinical studies, please specify allocation to treatment method); if randomization was used, please also state if restricted randomization was applied
- Indicate if masking was used during group allocation, data collection and/or data analysis

Please outline where this information can be found within the submission (e.g., sections or figure legends), or explain why this information doesn’t apply to your submission:

For kisspeptin-specific estrogen receptor knockout (KERKO) studies, as KERKO mice exhibited obvious phenotype, we did not perform blind randomization. Animals were grouped based on genotype.

For CRISPR-Cas9 based studies, animals that received different type of AAV vectors were randomized. At experimental endpoints, mice were then grouped based on the type of AAV vector carried single-guide RNA they received.

For *in vivo* measures, animals from different groups were mixed so any potential variation in the response of the animals due to time is normalized across all groups.

**Additional data files (“source data”)**

We encourage you to upload relevant additional data files, such as numerical data that are represented as a graph in a figure, or as a summary table

- Where provided, these should be in the most useful format, and they can be uploaded as “Source data” files linked to a main figure or table
- Include model definition files including the full list of parameters used
- Include code used for data analysis (e.g., R, MatLab)
- Avoid stating that data files are “available upon request”

Please indicate the figures or tables for which source data files have been provided:

A link to the code used for analysis of electrophysiological recordings has been provided in the Method session "*Data analysis and statistics”.*
